# Sagacious confucius’ pillow elixir ameliorates Dgalactose induced cognitive injury in mice *via* estrogenic effects and synaptic plasticity

**DOI:** 10.3389/fphar.2022.971385

**Published:** 2022-09-28

**Authors:** De-Ping Zhao, Xia Lei, Yue-Ying Wang, Ao Xue, Chen-Yu Zhao, Yan-Ming Xu, Yue Zhang, Guo-Liang Liu, Fang Geng, Hong-Dan Xu, Ning Zhang

**Affiliations:** ^1^ College of Pharmacy, Heilongjiang University of Chinese Medicine, Harbin, Heilongjiang, China; ^2^ Institute of Traditional Chinese Medicine, Wuxi Traditional Chinese Medicine Hospital, Jiangsu, Wuxi, China; ^3^ College of Jiamusi, Heilongjiang University of Chinese Medicine, Jiamusi, Heilongjiang, China; ^4^ Key Laboratory of Photochemistry Biomaterials and Energy Storage Materials of Heilongjiang Province, College of Chemistry and Chemical Engineering, Harbin Normal University, Harbin, Heilongjiang, China; ^5^ College of Pharmacy, Wuxi Higher Health Vocational Technology School, Wuxi, Jiangsu, China

**Keywords:** Sagacious Confucius’ Pillow Elixir, blood component, Alzheimer’s disease, estrogen receptor, synaptic plasticity

## Abstract

Alzheimer’s disease (AD) is a growing concern in modern society, and there is currently a lack of effective therapeutic drugs. Sagacious Confucius’ Pillow Elixir (SCPE) has been studied for the treatment of neurodegenerative diseases such as AD. This study aimed to reveal the key components and mechanisms of SCPE’s anti-AD effect by combining Ultra-high Performance Liquid Chromatography-electrostatic field Orbitrap combined high-resolution Mass Spectrometry (UPLC-LTQ/Orbitrap-MS) with a network pharmacology approach. And the mechanism was verified by *in vivo* experiments. Based on UPLC-LTQ/Orbitrap-MS technique identified 9 blood components from rat serum containing SCPE, corresponding to 113 anti-AD targets, and 15 of the 113 targets had high connectivity. KEGG pathway enrichment analysis showed that estrogen signaling pathway and synaptic signaling pathway were the most significantly enriched pathways in SCPE anti-AD, which has been proved by *in vivo* experiments. SCPE can exert estrogenic effects in the brain by increasing the amount of estrogen in the brain and the expression of ERα receptors. SCPE can enhance the synaptic structure plasticity by promoting the release of brain-derived neurotrophic factor (BDNF) secretion and improving actin polymerization and coordinates cofilin activity. In addition, SCPE also enhances synaptic functional plasticity by increasing the density of postsynaptic densified 95 (PSD95) proteins and the expression of functional receptor AMPA. SCPE is effective for treatment of AD and the mechanism is related to increasing estrogenic effects and improving synaptic plasticity. Our study revealed the synergistic effect of SCPE at the system level and showed that SCPE exhibits anti-AD effects in a multi-component, multi-target and multi-pathway manner. All these provide experimental support for the clinical application and drug development of SCPE in the prevention and treatment of AD.

## 1 Introduction

Alzheimer’s disease (AD) is a neurodegenerative disease associated with cognitive decline. The early stage of the disease is characterized by a lack of ability to encode new memories and store information, followed by cognitive and behavioral degeneration in the later stage (McKhann et al., 1984).

With the aging of the population, the number of AD patients will increase rapidly, which will bring increasingly serious social problems. The main pathological feature of AD is neuronal loss, and the hypothesis of an imbalance of cellular and molecular mechanisms underlying synaptic plasticity under neuronal loss is widely accepted (Mango et al., 2019). Synaptic plasticity refers to the ability of the brain to modify neural circuits in the structure and function of synapses to facilitate learning, memory, cognition, and emotional and behavioral regulation (Kennedy, 2013). Impaired synaptic plasticity is typically the loss of synaptic vesicles and major components of polypeptides, as well as extensive aberrant changes in the synapses themselves.

Dendritic spine shape, size or number has been altered in AD brain (Herms and Dorostkar, 2016) and transgenic AD mouse models (Spires et al., 2005; Jacobsen et al., 2006; Zou et al., 2015). At the same time, molecules involved in dendritic spine signaling (Sze et al., 2001; Mishizen-Eberz et al., 2004) and filamentous actin control were significantly reduced, resulting in severe synaptic dysfunction (Gordon-Weeks, 2016). Memory and learning deficits are highly correlated with changes in synaptic density beyond plaques or neurofibrillary tangles. Neuronal synaptic dysfunction already exists in the brain of AD patients in the early stages, which may be due to impaired ability of neurons to synthesize substances required for growth, inability of target cells to accept new synapses and/or changes in signaling thresholds. The degree of synaptic damage is positively correlated with the occurrence of AD cognitive impairment and other pathological mechanisms (Crimins et al., 2013; Fan et al., 2018; Barthet and Mulle, 2020). Therefore, synaptic biomarkers have been proposed as tools to study the link between AD molecular pathology and cognitive symptoms (Blennow and Zetterberg, 2015). Synaptic biomarkers are divided into presynaptic and postsynaptic markers. Presynaptic markers include those involved in calcium sensing and buffering such as parvalbumin, synaptotagmin, synaptophysin, etc., and postsynaptic markers include cytoskeletal proteins, growth/plasticity markers, and neurotransmitter receptors etc (Paasila et al., 2021). Decreased synaptic density and loss of synaptic proteins are present in AD patients, and anterior, posterior, and common synaptic markers are lost in multiple brain regions (Counts et al., 2006; Scheff et al., 2007; Paasila et al., 2021). Accumulating evidence suggests that synaptic plasticity plays an important role in AD pathology (Li et al., 2018; Chen et al., 2019; John and Reddy, 2021).

Epidemiological studies have demonstrated that the prevalence of AD has a gender tendency, and the prevalence of women is significantly higher than that of men in the proportion of the disease. Clinical experiments have shown that female patients with surgical removal of ovaries and patients with premature menopause have significantly reduced spatial memory and cognitive function, leading to an increased risk of AD, suggesting that the pathogenesis of AD may be related to estrogen deficiency (Hogervorst, 2013). A large number of studies have shown that estrogen in the brain can protect hippocampal neurons (Hu et al., 2003; Ishunina et al., 2007; Brooks et al., 2017), regulate synaptic plasticity in the hippocampus (Wang C. et al., 2016; Lu et al., 2019), and promote spatial memory behavior and learning ability (Benmansour et al., 2012). Estrogen in the brain mainly comes from the synthesis of ovarian organs and the conversion of neuron’s own androgens through aromatase, which mainly acts by binding to estrogen receptors and plays an important role in the regulation of the central nervous system (Gillies and McArthur, 2010). Through the removal of the ovaries and the application of aromatase inhibitors in mice, the synthesis of aromatase in the ovary is blocked and the activity of aromatase is specifically inhibited, and the level of estrogen is reduced. It is observed that the density of dendritic spines in the hippocampus decreases (Benmansour et al., 2016), learning and memory capability decline (Valdés and Weeks, 2010; Benmansour et al., 2014), and synaptic protein expression decreases (Dou et al., 2019). Therefore, it is inferred that estrogen affects synaptic plasticity by regulating the number of dendritic spines and the expression of functional proteins in the synaptic membrane. Studies have found that estradiol replacement therapy (ERT) has potential value in AD treatment and prevention, but the long-term application of this therapy will increase the risk of breast cancer, endometrial cancer and other diseases (Nie et al., 2013). Therefore, finding and developing safe and estrogen-modulating anti-AD drugs is an important research area.

There are many pathogenic mechanisms of AD, and Traditional Chinese Medicine (TCM) alone or in combination has been shown to improve memory impairment and quality of life in AD patients (Lee J. E. et al., 2020; Fu et al., 2020). In addition, Chinese herbal formulas are widely used in clinical practice due to their multi-component and multi-target treatment of Alzheimer’s disease (Wang Z. Y. et al., 2016; Yang et al., 2017). Sagacious Confucius’ Pillow Elixir (SCPE) is a representative formula with the effect of invigorating the kidney and filling the essence, including *Polygala tenuifolia Willd.*, *Acorus tatarinowii Schott*, *Chinemys reevesii(Gray)*, *Os Draconis*. Its traditional effect is to invigorate the kidney, soothe the nerves, improve learning and memory ability, and it is widely used in modern Chinese medicine to treat AD (Chen et al., 2017; Hou et al., 2019; Jin, 2019; Lou et al., 2019; Sheng et al., 2020) Some ingredients in the formula have a certain role in improving AD. For example, polygala saponins can inhibit neuroinflammation, reduce neural excitotoxicity, increase synaptic plasticity, alleviate Aβ pathology and inhibit neuronal death by promoting mitophagy inhibition, resulting in neuroprotective effects (Qiu et al., 2022; Sun et al., 2022); paracoumarin can protect cells from Aβ neurotoxicity and reduce ROS accumulation (Hong et al., 2012); β-asarone can reduce Bcl-2 expression and achieve neuroprotective effect (Wei et al., 2013; Chen et al., 2014; Liu et al., 2016). However, due to the complex composition of SCPE and the characteristics of multiple targets and multiple pathways, the underlying mechanism of its active components’ anti-AD effect is still unclear. Therefore, UPLC-LTQ-Orbitrap-MS combined with network pharmacology method was used to study the active components of SCPE and its anti-AD mechanism, which provided a new perspective for the study of traditional Chinese medicine formulations (Zineh, 2019). To our knowledge, this is the first study on UPLC-LTQ-Orbitrap-MS combined with network pharmacology analysis to explain the chemical signature of SCPE’s anti-AD effect.

**FIGURE 1:**
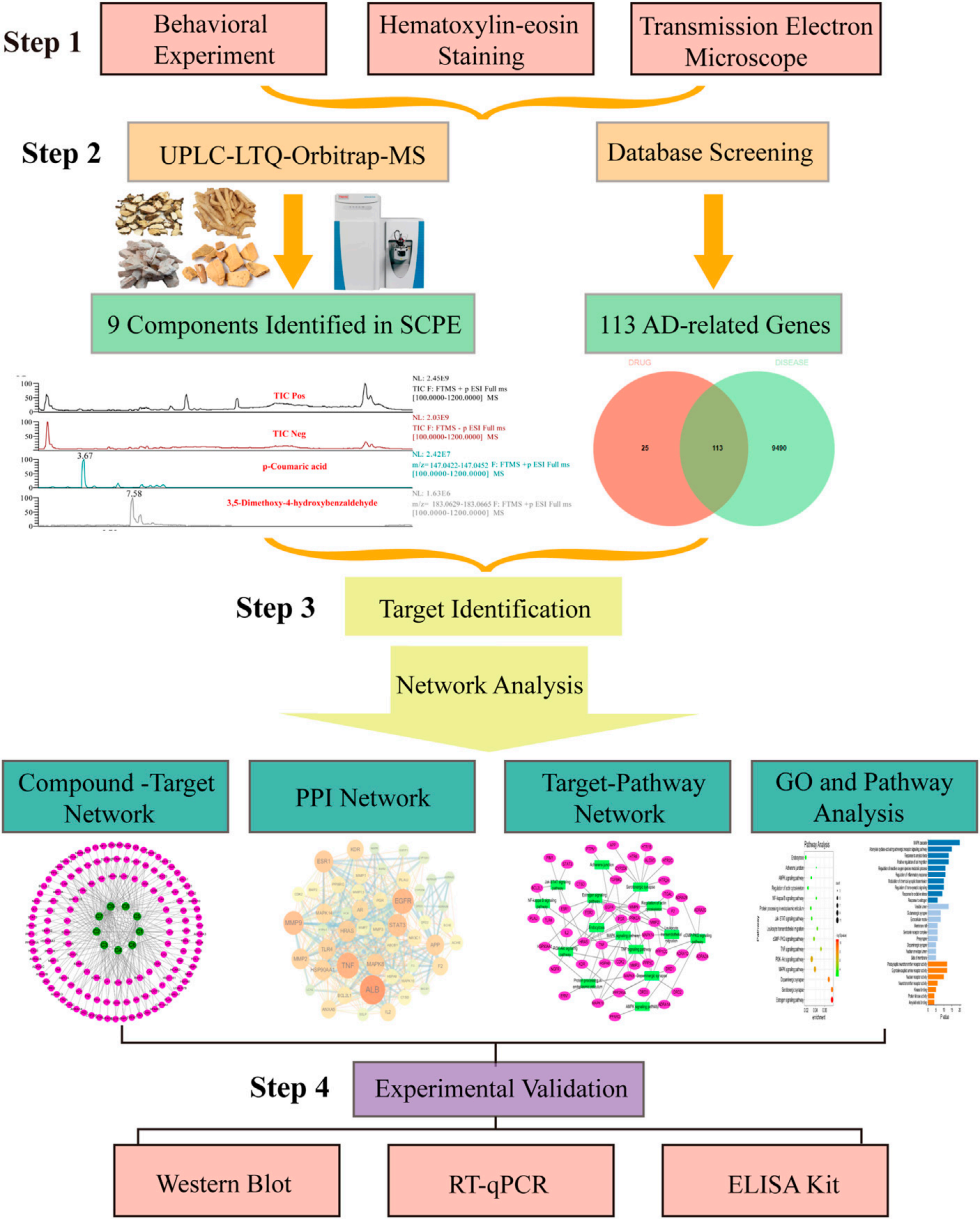
Flow of experimental research of SCPE in the treatment of Alzheimer’s disease. Step-by-step graphical representation of the experimental workflow.The initial step in this process is to demonstrate the effectiveness of SCPE. Followed by a second step, which is the analysis of active ingredients of SCPE by UPLC-LTQ-Orbitrap-MS. Based on the ingredients found in previou step, the third step is combined with network pharmacology to find the potential anti-AD mechanism of SCPE. Step 4 Use molecular biology techniques verify Experiment. The final step in this experiment is to experimentally verify the anti-AD effect of SCPE.

In the current study, we performed a series of experimental analyses in Kunming mice to confirm that SCPE can improve memory cognitive impairment and restore neuronal morphology in Dgalactose model mice. On this basis, we identified the potential bioactive components in SCPE, and performed a series of network pharmacology analyses using the blood component, including target prediction and enrichment, pathway analysis and network construction, to identify SCPE and AD-related targets and underlying mechanisms. Guided by the results of network pharmacology, we verified the mechanism by which SCPE exerts neuroprotective effects. These mechanisms include that SCPE increases the level of estrogen in the brain and the expression of ERα receptors, exerts estrogenic effects in the brain, promotes the secretion of BDNF, and regulates the structural plasticity and functional plasticity of neurons in the hippocampal CA1 region. Workflow of our network pharmacology and experimental study of SCPE in AD is shown in Figure 1. Our results not only demonstrate the synergistic anti-AD activity of SCPE and its potential targets, but also provide insight into the mechanisms at the molecular biological level of SCPE.

## 2 Materials and methods

### 2.1 Drugs

SCPE is our laboratory-made. *Polygala tenuifolia Willd.*, *Acorus tatarinowii Schott*, *Chinemys reevesii(Gray)*, *Os Draconis* are provided in Beijing Tong Ren Chinese Medicine co.Ltd. Drug Store (Harbin, China). Appraised by Associate Professor Chen Xiaozhong of Heilongjiang University of Traditional Chinese Medicine, it meets the requirements of the 2020 edition of the Chinese Pharmacopoeia. Mix according to the ratio of 1:1:1:1, decoct and concentrate to 1 g/ml for later use. Dgalactose was provided by Macklin Co., Ltd. (Beijing, China); Estradiol was provided by Bayer Pharma Co., Ltd. (Leverkusen, Germany).

### 2.2 Animals and treatment

12 weeks Kun Ming (KM) mice (25 ± 5 g) and 8 weeks Sprague Dawley (SD) rats (230 ± 20 g) were purchased from Liaoning Changsheng Biotechnology Co., Ltd. (Liaoning, China). All animals were housed in a barrier system with adjustable temperature (21 ± 2°C) and humidity (50 ± 5%) with a light-dark cycle of 12 h/d. All animal studies were performed in accordance with institutional guidelines for the care and use of laboratory animals, and the protocol was approved by the Animal Research Ethics Committee of Heilongjiang University of Traditional Chinese Medicine.

SD rats were randomly divided into Control group and SCPE group (6 rats per group). Control group was given distilled water by gavage, and SCPE group was given 5.4 g/kg SCPE diluent. After 0.5, 1, 1.5, and 2 h, the orbital blood was collected, and the mice were centrifuged (4 °C, 12,000 rpm, 30 min) after standing for 30 min, and take the serum and store it in a -80°C refrigerator. KM mice were randomly divided into four groups (15 KM mice per group) for various treatments, including Control group (normal saline); Model group (Dgalactose, 200 mg/kg) (Chen et al., 2016); SCPE group (SCPE with 7.8 g/kg); Postive group (Estradiol, 1.3 mg/kg), all animals dosed in a volume of 0.2 ml. After animal behavior, the mice were anesthetized by 2% isoflurane inhalation anesthesia, and the respiratory rate and lower limb muscle reflex of the mice were observed. After the respiratory rate of the mice slowed down and the reflexes weakened, the orbital blood of the mice was collected for ELISA detection. The mice heart was perfused with normal saline, and the bilateral hippocampus was stripped on ice and placed in 2.5% glutaraldehyde fixative for electron microscopy to detect the ultrastructure of the hippocampus; the hippocampus was stored in liquid nitrogen for Western Blot and RT-qPCR experiments. The mice were perfused with 4% paraformaldehyde, and whole brains were taken for H and E staining.

### 2.3 Behavioral experiments

Morris water maze (MWM) experiment was carried out using the method reported by Shi et al. (Du et al., 2020), and the learning and memory abilities of spatial location sense and spatial orientation were respectively investigated by positioning navigation and spatial exploration experiments. According to the method proposed by Luo et al. (2019). (Luo et al., 2019), novel object recognition (NOR) experiment was carried out. The mice were placed in the same position, and the contact time of the mice to the new object (Tn) and the contact time of the old object (Tf) within 5 min were recorded. Recognition index=(Tn-Tf)/(Tn+Tf) was used to represent the recognition ability of mice to objects. The level of recognition index represents the strength or weakness of the memory ability of mice.

### 2.4 Hematoxylin and eosin staining

First, the brain tissue to be tested was fixed with 4% paraformaldehyde for 24 h, dehydrated with graded ethanol, embedded in paraffin with a melting point of 56°C, and sliced with a paraffin microtome. The brain tissue sections were then dewaxed with xylene, 95% ethanol, 90% ethanol, and 85% ethanol, and then placed in distilled water for staining. Brain tissue sections were stained with hematoxylin for 8 min, the excess dye was washed away with distilled water, and then placed in acidic alcohol for 30 s differentiation. Sections were washed with distilled water, then immersed in 1% eosin aqueous solution for 1 min, and the excess eosin was washed off. Gradient alcohol, xylene dehydration, and cover slip. Observation of pathological changes of mouse hippocampus under light microscope.

### 2.5 Transmission electron microscopy

The hippocampal tissue block was cut into about 1 mm^3^, placed in 2.5% glutaraldehyde for 2 h, fixed with 1% fixative for 1.5 h, dehydrated, infiltrated, embedded and sliced, and stained with 2% toluidine blue for localization. Ultrathin sections were placed under a 200-mesh copper mesh for double staining (uranyl acetate-lead citrate), and the ultrastructural changes of mouse hippocampal neurons were observed and photographed under electron microscope.

### 2.6 Ultra-high performance liquid chromatography-electrostatic field Orbitrap combined high-resolution mass spectrometry

Take 500 μL serum samples of SD rats into a centrifuge tube, add 2 ml methanol, 50 μL acetic acid solution, vortex for 1 min, sonicate for 1 min, and then centrifuge (13,000 rpm, 10 min), take the supernatant, blow with nitrogen. Reconstituted with 100 μL of 70% methanol solution, passed through a 0.22 μm microporous membrane (Millipore, United States), and injected 5 μL.

UPLC was performed on an UltiMate 3,000 system (Thermo, United States) using an ACQUITY UPLC HSS T3 column (100 mm × 2.1 mm, 1.8 μm) for chromatographic separation. Column and autosampler temperatures were respectively maintained at 40°C and 4°C. The mobile phases were solvent A (0.1% formic acid-water) and B (1% formic acid-acetonitrile). The optimized gradient elution program was flow rate: 0–3.5 min, 0%–15% B; 3.5–6 min, 15 %–30% B; 6–12 min, 30%–70% B; 12–12.5 min, 30%–70% B; 12.5–18 min, 100% B. The flow rate was 0.4 ml/min and the injection volume was 5 µL. MS analysis was performed using an LTQ-Orbitrap system (Thermo, United States) with an electrospray ionization source (ESI) operating in positive and negative ion mode. The spray voltage is 3.5 kV (positive ion mode) and 3.2 kV (negative ion mode), ion transfer tube temperature: 320 °C; AUG gas heating temperature: 350 °C; collision energy: 20 eV, 40 eV, 60 eV; sheath gas: 40 psi; auxiliary gas: 20 psi; purge gas: 10 psi; RF lens amplitude (s-lens): 60; resolution: primary high resolution mass spectrometry 70000 FWHM, secondary high resolution mass spectrometry 17500 FWHM; s; range m/z 50–1,500. The obtained data were processed with Thermo Scientific Xcalibur (Thermo, United States) software, and the compounds were analyzed by retention time, exact molecular mass, elemental composition, primary mass spectrum (5 ppm error), secondary mass spectrum (10 ppm error) and fragmentation The rules are compared with the composition database to determine the chemical composition.

### 2.7 Network pharmacology analysis

Biological targets were predicted using all influx components in the chemical profile in SCPE identified by UPLC-LTQ-Orbitrap-MS. Use ChemBioDraw software (http://www.ChemBioDraw) to draw the three-dimensional chemical structure of each component in SDF format. Imported into PharmMapper (http://www.lilab-ecust.cn/Pharmmapper/) and the Swiss Target Prediction database (http://www.swisstargetprediction.ch/) for prediction of organisms with “*Homo sapiens*” the UniProt ID of the target.

Information on Alzheimer’s disease-related targets was collected from the following four databases: Therapeutic Target Database (http://bidd.nus.edu.sg/group/cjttd/), Disgenet Database (https://www. disgenet.org), DrugBank database (https://www.drugbank.ca/) and GeneCards (https://www.genecards.org/). Select “Alzheimer’s disease” as the keyword, limit the species to “*Homo sapiens*”, and collect targets that are repeated more than twice.

After the targets related to the identified components of SCPE and the anti-AD related targets were crossed, the overlapping targets were screened out as potential targets. The components directly related to Alzheimer’s disease were screened out as potential bioactive components. Then, compound-target interaction networks were generated using Cytoscape v3.7.1, a powerful bioinformatics software package for integrating, analyzing, and visualizing biomolecular interaction networks.

The targets of the key components in SCPE obtained from the network pharmacology analysis were uploaded to the STRING database for protein-protein interaction (PPI) analysis. The study species was defined as “*Homo sapiens*”, and the remaining parameters were set to default settings to obtain the PPI network. Target genes with high intermediateness and compactness were selected as key genes for SCPE anti-AD.

Targets selected from the above analyses were uploaded to the Matespace database (https://metascape.org/) for analysis of GO enrichment and KEGG pathway analysis to obtain pathway information. The *P* value was tested using the FDR error control method, and the threshold was set to *p* < 0.01. Target-pathway interaction networks were generated using Cytoscape v3.7.1 to provide information on interactions between active compounds, targets, pathways, and AD-related pathways.

### 2.8 Western blot analysis

The hippocampus was placed in RIPA buffer (Cell Signaling Tech, United States) containing a cocktail of protease and phosphatase inhibitors. Protein levels in the lysates were determined using the BCA protein assay kit (Bio-Rad, United States). Equal amounts of protein were loaded into each channel of a sodium dodecyl sulfate-polyacrylamide gel electrophoresis double gel and then electrophoretically transferred to a polyvinylidene fluoride (PVDF) membrane (Millipore, United States). Membranes were washed with Tris-buffered saline containing 0.1% Tween 20 (TBST) and then blocked in buffer containing 5% nonfat dry milk for 120 min. After washing three times with TBST, the primary antibody was incubated overnight in a 4°C refrigerator. ERα (1:1,000; cat. no. PB0188; Boster, China), BDNF (1:1,000; cat. no. PB9075; Boster, China), cofilin (1:500; cat. no. PB9033; Boster, China), P-cofilin (1:500; cat. no. 3311; Cell Signaling Tech, United States), PSD95 (1:500; cat. no. BA3304; Boster, China), AMPA (1:1,000; cat. no. ab32436; abcam, UK), GAPDH (1:1,000; cat. no. BM1623; Boster, China).The PVDF membrane was further washed three times with TBST, and the secondary antibody was incubated for 60 min at room temperature. HRP-labeled goat anti-rabbit IgG (1:5000; cat. no. BM3896; Boster, China). Immune complexes were detected with enhanced chemiluminescence (ECL) detection reagent, and protein levels were quantified with ImageJ.

### 2.9 RT-qPCR analysis

Total RNA from hippocampal tissue was extracted according to the manufacturer’s protocol, and cDNA was synthesized with random primers using a high-capacity cDNA reverse transcription kit using total RNA (200 ng) as a template. All PCR reactions were performed using SGExcel UltraSYBR Mixture (with ROX) (Sangon Biotech Co., Ltd., Shanghai, China) to a final volume of 20 μL. Each cDNA sample was run in triplicate in the Mx3000P Sequence Detection System (Agilent, United States). Primers were designed by Primer Premier software and synthesized by Sangon Biotech Co., Ltd. (Shanghai, China). The following additional primers were used:

CYP19: forward 5′-TCA​TGA​AGC​ACA​GTC​ACT​ACA​T-3′, reverse 5′-AAA​CTT​CCA​CCA​TTC​GAA​CAA​G-3';

GAPDH: forward 5′-CTA​CCT​CAT​GAA​GAT​CCT​GAC​C-3′, reverse 5′-CAC​AGC​TTC​TCT​TTG​ATG​TCA​C-3'.

### 2.10 G-actin/F-actin ratio measurement

Soluble G-actin was separated from filamentous F-actinusing the G-Actin/F-Actin *In Vivo* Assay Kit (cat. no.BK037, Cytoskeleton, Inc., Denver, CO, United States) according to the manufacturer’s instructions. Briefly, hippocampal tissue was triturated in liquid nitrogen and then added with LAS2 buffer, then F-actin-enhancing solution was added to the sample at a volume ratio of 1:100, left to stand for 10 min, centrifuged (2,000 rpm, 5 min). Take the supernatant and centrifuge again (100,000×g, 37°C, 1 h) to precipitate F-actin. Separate the supernatant containing G-actin and add F-actin depolymerization buffer to the precipitate, place on ice for 1 h, and invert the sample every 15 min to obtain the G-actin solution produced by F-actin depolymerization. Finally, add 5x SDS sample buffer to each tube and assess the G-actin/F-actin ratio using western blotting.

### 2.11 ELISA kits analysis

Estradiol (E2) content in hippocampal lysates was measured using an E2 content kit (Elabscience, China).

### 2.12 Statistical analysis

The data results were analyzed and processed by SPSS 21.0 (SPSS Inc., Chicago, IL, United States) statistical software, the data were expressed as mean ± standard deviation (mean ± SD), and the comparison between groups was processed by one-way ANOVA (one-way ANOVA).), *p* < 0.05 indicates statistical significance.

## 3 Results

### 3.1 Effect of SCPE on the learning and memory ability and histomorphology of model mice

**FIGURE 2:**
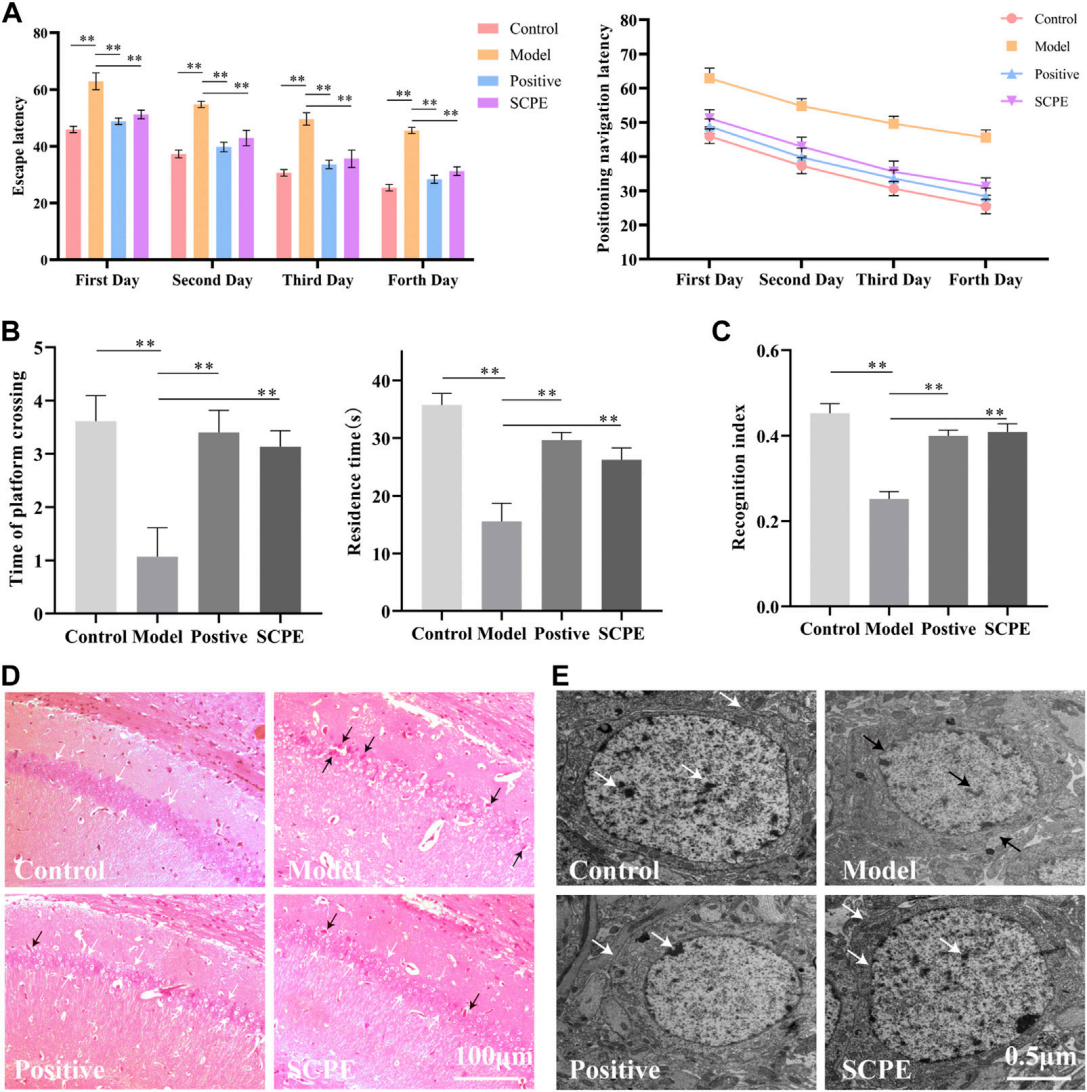
Analysis of SCPE on learning and memory ability and histomorphology of model mice. **(A)** Positioning navigation latency. **(B)**Time of platform crossing and residence time. **(C)**Recognition index. **(D)**H and E staining × 200. **(E)**Transmission electron microscope × 22000. Data in **(A–C)** presented as the mean ± SD of fifteen independent experiments. Data in **(D–E)** presented as the mean ± SD of three independent experiments.*p < 0.05, **p < 0.01 compared with indicated groups. HE, hematoxylin and eosin.

The improvement effect of SCPE on the learning and memory ability of Dgalactose model mice was evaluated by MWM and NOR experiments.The results showed that compared with Control group, the latency of Model group was significantly increased; compared with Model group, Postive group and SCPE group were significantly decreased in latency. The platform-seeking latency of the four groups of mice gradually decreased during the 4-day training, but the latency of Control group, Postive group and SCPE group decreased rapidly, and the latency of the Model group decreased less (Figure 2A, Table 1). Compared with Control group, the number of crossing platforms and the dwell time in the target quadrant were significantly reduced in Model group; compared with Model group, the number of crossing platforms and the dwell time in the target quadrant were significantly increased in Postive group and SCPE group (Figure 2B, Table 2). Compared with Control group, the new object recognition index of Model group mice was significantly reduced; Recognition index increased significantly in Positive group and SCPE group (Figure 2C, Table 3). The trajectory of the mouse to find the platform (Supplementary Figure S1).

**TABLE 1:** Effect of SCPE on the escape latency of D-galactose model mice (mean ± SD, *n* = 15).

Group	First day	Second day	Third day	Forth day
Control	45.987 ± 1.119	37.358 ± 1.339	30.686 ± 1.106	25.435 ± 1.127
Model	62.946 ± 2.944^**^	54.837 ± 1.107^**^	49.667 ± 2.163^**^	45.609 ± 1.106^**^
Positive	48.865 ± 1.154^##^	39.822 ± 1.664^##^	33.629 ± 1.502^##^	28.412 ± 1.413^##^
SCPE	51.220 ± 1.528^##^	42.950 ± 2.752^##^	35.629 ± 3.085^##^	31.258 ± 1.512^##^
F value	48.918	53.844	48.184	141.608
*P* value	0.001	0.001	0.001	0.001

Note: ^**^ *p* < 0.01, compared with Control group; ^##^ *p* < 0.01, compared with Model Group.

**TABLE 2:** Effect of SCPE on space exploration of D-galactose model mice (mean ± SD, *n* = 15).

Group	Time of crossing platforms	Residence tine(s)
Control	3.267 ± 0.884	35.741 ± 1.995
Model	1.133 ± 0.743^**^	15.570 ± 3.109^**^
Positive	3.067 ± 0.799^##^	29.673 ± 1.296^##^
SCPE	2.733 ± 0.704^##^	26.253 ± 2.035^##^
F value	20.771	120.797
*P* value	0.001	0.001

Note: ^**^ *p* < 0.01, compared with Control group; ^##^ *p* < 0.01, compared with Model Group.

**TABLE 3:** Effect of SCPE on the new object recognition experiment of D-galactose model mice (mean ± SD, *n* = 15).

Group	Recognition index
Control	0.453 ± 0.022
Model	0.252 ± 0.017^**^
Positive	0.400 ± 0.013^##^
SCPE	0.409 ± 0.019^##^
F value	70.437
*P* value	0.001

Note: ^**^ *p* < 0.01, compared with Control group; ^##^ *p* < 0.01, compared with Model Group.

H and E staining light microscope observed that the cells in the hippocampus CA1 regions of Control group mice were regularly arranged, the number of neurons were large, the staining was uniform, the nucleoli were clear, and there were no nuclear pyknosis. The hippocampal cells of Model group mice are disordered, some neurons are lost, the cells are shrunk, the nucleoli are not obvious, and the phenomenon of pyknosis is obvious. Positive group, SCPE group and Control group are similar (Figure 2D).

Observation of mouse hippocampus by transmission electron microscope showed that the structure of nuclear membrane of neurons in Control group, Positive group and SCPE group were clear, and the chromatin was like fine sand. The nucleus occupies most of the cell, the rough endoplasmic reticulum is abundant in the cytoplasm, the structure of the Golgi apparatus is normal, there are many free nucleoprotein bodies, the Nissl bodies are clearly visible, and there are occasional plasmin granules. The synaptic membrane structure of the neuropil area is intact and the number of vesicles is high. The hippocampal neurons under the microscope of the Model group are the opposite of the above three groups (Figure 2E). This indicates that SCPE has a certain neuroprotective effect.

### 3.2 Identification of SCPE blood components

**FIGURE 3:**
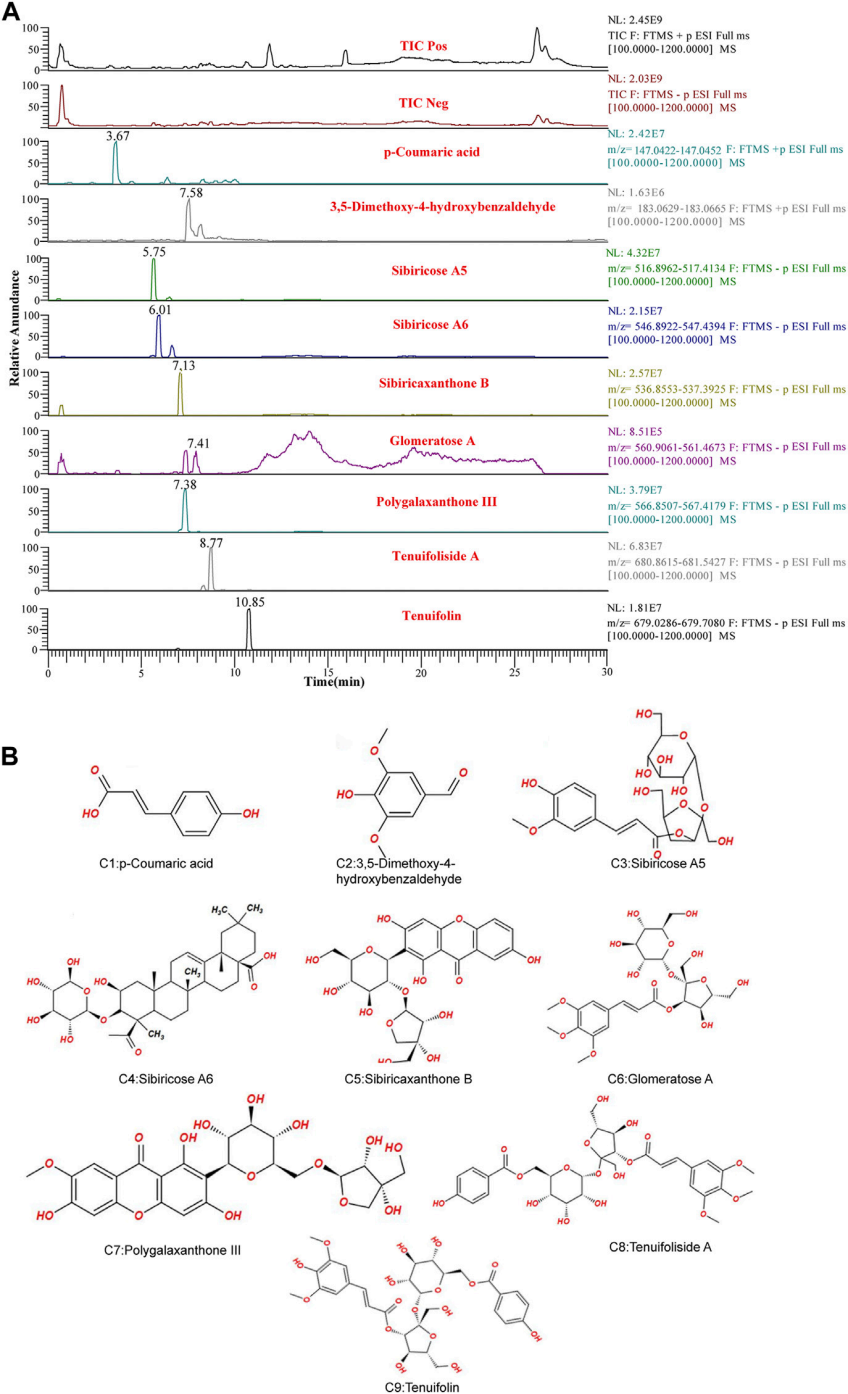
UPLC-LTQ-Orbitrap-MS spectrometry of SCPE serum characteristic compounds. **(A)** Total ion chromatogram (TIC) and selected ion chromatogram (SIC) of SCPE influent sample. Stationary Phase: ACQUITY UPLCTM HSS T3 (100 mm × 2.1 mm, 1.8 μm); Mobile phase: Solvents A (0.1% formic acid-water) and B (1% formic acid-acetonitrile). Optimized gradient elution program: 0–3.5 in, 0%–15% B; 3.5–6 min, 15%–30%B; 6–12 min, 30%–70% B; 12–12.5 min, 30%–70% B; 12.5–18 min, 100% B; Flow rate: 0.4 ml/min (Peak number: Rt,3.67 min, p-Coumaric acid; 7.58 min, 3,5-Dimethoxy-4-hydroxybenzaldehyde; 5.75 min, Sibiricose A5; 6.01 min, Sibiricose A6; 7.13 min, Sibiricaxanthone B; 7.41 min, Glomeratose A; 7.38 min, olygalaxanthone III; 8.77 min, Tenuifoliside A; 10.85 min, Tenuifolin). **(B)** Molecular formulas of 9 blood-transporting active compounds identified from SCPE.

UPLC-LTQ-Orbitrap-MS was used to qualitatively analyze the whole prescription of SCPE, and the total ion chromatograms of blank serum and drug-containing serum were compared. Perform operations such as peak matching, peak alignment, and peak extraction according to retention time and mass-to-nucleus ratio to obtain a three-dimensional matrix of retention time, mass-to-nucleus ratio, and peak area. Finally, the presence or absence of the peak area was used to determine whether the ion was a unique component in the drug-containing serum. Nine blood-introducing prototype components were determined by the fragmentation rule of the mass-nucleus ratio fragments and database comparison. The 9 blood prototype components include 2 phenolic acid components: p-Coumaric acid (C1), 3,5-Dimethoxy-4-hydroxybenzaldehyde (C2); four oligosaccharide ester components: Sibiricose A5 (C3), Sibiricose A6 (C4), Glomeratose A (C6), Tenuifoliside A (C8); 2 xanthones components: Sibiricaxanthone B (C5), Polygalaxanthone III (C7); 1 saponin component: Tenuifolin (C9). The ion current diagram of the component entering the blood is shown in Figure 3A, the structure is shown in Figure 3B, and the information is shown in Table 4.

**TABLE 4:** UPLC-MS characterization of the main components of SCPE

NO	Compound	Chemical formula	m/z	Accuracy (ppm)	Adduct method	RT (min)
1	p-Coumaric acid	C_9_H_8_O_3_	147.0437	−1.82	[M+H-H2O]^+^	3.67
2	3,5-Dimethoxy-4-hydroxybenzaldehyde	C_9_H_10_O_4_	183.0647	−2.64	[M+H]^+^	7.58
3	Sibiricose A5	C_22_H_30_O_14_	517.1548	−2.71	[M-H]^-^	5.75
4	Sibiricose A6	C_23_H_32_O_15_	547.1658	−1.87	[M-H]^-^	6.01
5	Sibiricaxanthone B	C_24_H_26_O_14_	537.1239	−1.85	[M-H]^-^	7.13
6	Glomeratose A	C_24_H_34_O_15_	561.1867	−1.99	[M-H]^-^	7.41
7	Polygalaxanthone III	C_25_H_28_O_15_	567.1343	−2.1	[M-H]^-^	7.38
8	Tenuifoliside A	C_31_H_38_O_17_	681.2021	−2.19	[M-H]^-^	8.77
9	Tenuifolin	C_36_H_56_O_12_	679.3683	−2.36	[M-H]^-^	10.85

### 3.3 Network pharmacology

#### 3.3.1 Compound-target network analysis

**FIGURE 4:**
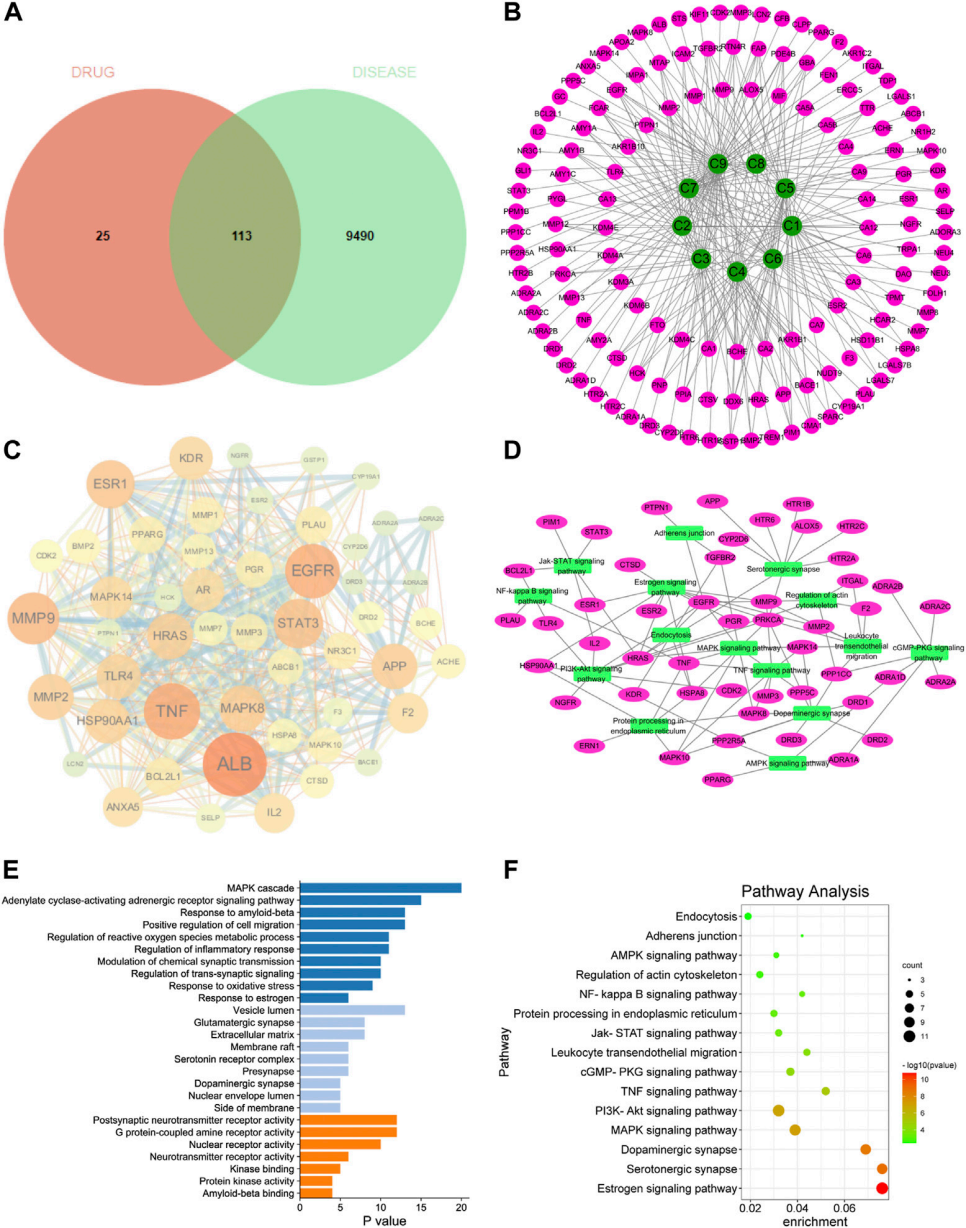
Network pharmacology analyze. **(A)** Venn diagram of SCPE-related targets and AD-related targets; **(B)** Compound-target network of SCPE and AD. Green nodes represent candidate active compounds and pink nodes represent potential protein targets. Edges represent the interactions between them. **(C)** Protein-protein interaction (PPI) network of SCPE-related targets in AD. The size of the node is proportional to the degree of the node. **(D)** Target-pathway network of SCPE. Green nodes represent paths, pink nodes represent targets, and edges represent interactions between them. **(E)**List of Gene Ontology (GO) results in relation to the potential targets of SCPE.The first 26 GO terms were identified based on *p* < 0.01.**(F)**Kyoto Encyclopedia of Genes and Genomes (KEGG) pathway enrichment results in relation to the potential targets of SCPE.The top 15 pathways were identified based on *p* < 0.05.

In order to screen the potential anti-AD components of SCPE and study its mechanism of action, we used network pharmacology techniques to predict its potential active components and targets. First, by using the TTD, Disgent, GeneCards, DrugBank, Pharmapper, and SwissTargetPrediction databases, we respectively obtained 138 targets and 9603 AD-related targets among the 9 identified blood-penetrating compounds. After intersecting component targets with AD-related targets, 113 overlapping targets were screened as potential AD-related targets (Figure 4A). The 113 potential targets and 9 compounds were entered into Cytoscape software to visualize compound-target networks. Oligosaccharide ester compounds acted on 51 targets, and xanthones components acted on 38 targets (Figure 4B).

#### 3.3.2 PPI network of SCPE-related AD targets

To further explore the importance of the 113 targets acting on the nine key components, potential targets were uploaded to the STRING database and protein-protein interactions (PPI) were further constructed using Cytoscape software. In the PPI network, *ALB*, *EGFR*, *TNF*, *MMP9*, *ESR1*, *STAT3*, *APP*, *MMP2*, *HSP90AA1*, *HRAS*, *MAPK14*, *TLR4*, *KDR*, *MAPK8*, *CYP19A1* had higher connectivity in the PPI analysis (Figure 4C **).**

#### 3.3.3 GO and KEGG pathway enrichment analysis

Potential targets of SCPE for AD treatment were subjected to GO enrichment analysis as well as KEGG pathway analysis to understand the relationship between functional units and their potential significance in biological system networks. The biological processes of GO are mainly MAPK cascade, Modulation of chemical synaptic transmission, Regulation of trans-synaptic signaling, and Response to estrogen. Regarding GO cell components, it mainly includes Vesicle lumen, Glutamatergic synapse, membrane raft, Serotonin receptor complex, Presynapse, and Dopaminergic synapse. In terms of GO molecular functions, they were mainly enriched in Postsynaptic neurotransmitter receptor activity, G protein-coupled amine receptor activity, and Neurotransmitter receptor activity (Figure 4E **).**

A target-pathway network of nine key components was constructed using Cytoscape software (Figure 4D–F). All five of the 113 key pathways involved ten key targets: estrogen signaling pathway (associated with key targets *ESR1*, *EGFR*, *MMP9*, *HRAS, HSP90AA1*), serotonergic synapses (associated with key targets *HTR6*, *APP*, *HRAS*), dopaminergic synapses (related to key targets *DRD1*, *MAPK14*), MAPK signaling pathways (related to key targets *MAPK14*, *EGFR*, *HRAS*, *MAPK8*) and actin cytoskeleton regulation (related to key targets *EGFR*, *HRAS*, *HSP90AA1*). These data suggest that the therapeutic mechanism of SCPE for AD may be achieved through these targets and signaling pathways. SCPE acted on AD in a multi-path, multi-objective and holistic cooperative manner.

### 3.4 Effects of SCPE on estrogen levels in Dgalactose model mice

#### 3.4.1 Serum E2 and CYP19 mRNA expression levels

**FIGURE 5:**
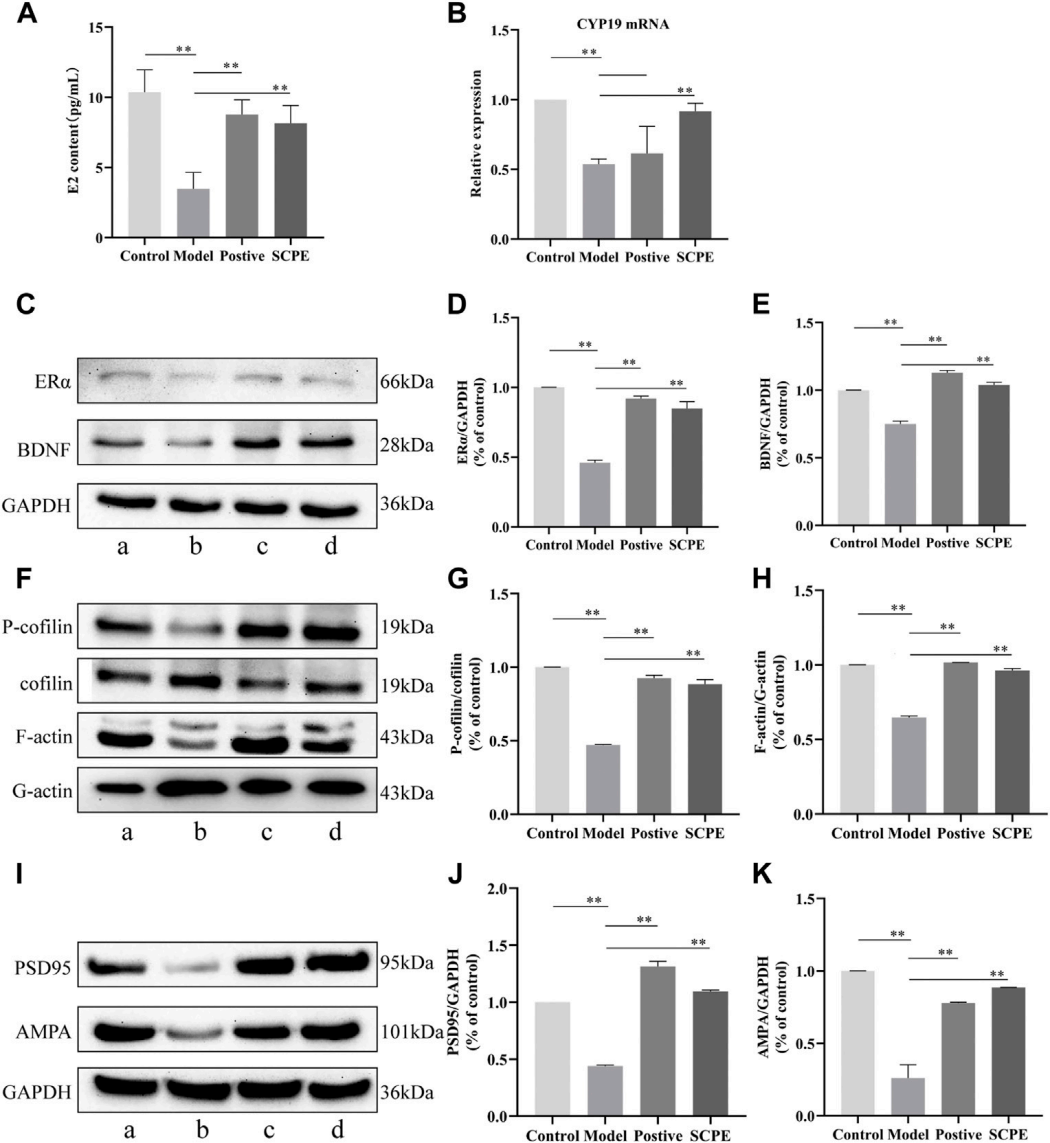
Effects of SCPE on estrogen and synaptic plasticity in the hippocampus.**(A)** Determination of E2 content in mouse hippocampus by ELISA kit. **(B)** RT-qPCR analysis of CYP19 mRNA expression in mouse hippocampus. **(C) (F) (I)** Proteins in the mouse hippocampus were analyzed by Western blot.a: Control group; **(B)** Model group; **(C)** Postive group; **(D)** SCPE group. **(D,E) (G,H) (J,K)** Relative expression levels analyzed with ImageJ software are shown. Data in **(A–K)** presented as the mean ± SD of three independent experiments. *p < 0.05, **p < 0.01 compared with indicated groups.

To investigate whether SCPE can increase estrogen in the brain, we assessed the effect of SCPE on estrogen in the brain by ELISA kit and RT-qPCR analysis. Similar results appeared between Control group, SCPE group and Postive group, with significantly increased E2 levels in mice compared with Model group (*p* < 0.01). Compared with the Model group, the Control group and SCPE group had significantly increased *CYP19 mRNA* expression (*p* < 0.01). Interestingly, we found that the expression of *CYP19 mRNA* in Postive group mice did not increase significantly compared with Model group (Figures 5A,B, Table 5, 6).

**TABLE 5:** E_2_ content in mice (mean ± SD, *n* = 3).

Group	E_2_ content (pg/mL)
Control	10.36 ± 1.60
Model	4.49 ± 2.16^**^
Positive	8.78 ± 1.04^##^
SCPE	8.15 ± 1.27^##^
F value	15.859
*P* value	0.001

Note: ^**^ *p* < 0.01, compared with Control group; ^##^ *p* < 0.01, compared with Model Group.

**TABLE 6:** Effect of SCPE on *CYP19 mRNA* expression in the hippocampus of D-galactose model mice (mean ± SD, *n* = 3).

Group	2^-△△ct^
Control	1.000 ± 0.000
Model	0.537 ± 0.037^**^
Positive	0.614 ± 0.194
SCPE	0.893 ± 0.089^##^
F value	14.448
*P* value	0.001

Note: ^**^ *p* < 0.01, compared with Control group; ^##^ *p* < 0.01, compared with Model Group.

#### 3.4.2 The effect of SCPE on the indexes of ERα-BDNF pathway in the hippocampus of Dgalactose model mice

Compared with the Control group, the expressions of ERα and BDNF proteins in the hippocampus of the Model group mice were significantly decreased (*p* < 0.01). Positive group and SCPE group have similar trends to Control group (*p* < 0.01) **(** Figures 5C–E, Table 7).

**TABLE 7:** Protein expression of ERα and BDNF in hippocampus of D-galactose model mice by SCPE (mean ± SD, *n* = 3).

Group	ERα/GAPDH	BDNF/GAPDH
Control	1.000 ± 0.0000	1.000 ± 0.0000
Model	0.460 ± 0.0191^**^	0.7514 ± 0.0203^**^
Positive	0.9207 ± 0.0167^##^	1.1285 ± 0.0178^##^
SCPE	0.8498 ± 0.0483^##^	1.0375 ± 0.0207^##^
F value	231.818	269.65
*P* value	0.001	0.001

Note: ^**^ *p* < 0.01, compared with Control group; ^##^ *p* < 0.01, compared with Model Group.

### 3.5 Effects of SCPE on hippocampal synapses in Dgalactose model mice

#### 3.5.1 The effect of SCPE on the plasticity of hippocampal synapse in Dgalactose model mice

We next explored the effect of SCPE treatment on synaptic structural plasticity in Dgalactose model mice. We assessed the expression of F-actin/G-actin and P-cofilin/cofilin by western blotting. Compared with Model group, the expression levels of F-actin/G-actin and P-cofilin/cofilin were significantly increased in Control group, Postive group and SCPE group (*p* < 0.01) (Figures 5F–H, Table 8).

**TABLE 8:** Protein expression of P-cofilin/cofilin and F-actin/G-actin in mouse hippocampus (mean ± SD, *n* = 3).

Group	P-cofilin/cofilin	F-actin/G-actin
Control	1.000 ± 0.0000	1.000 ± 0.0000
Model	0.4694 ± 0.0046^**^	0.6473 ± 0.0097^**^
Positive	0.9262 ± 0.01835^##^	1.0158 ± 0.0008^##^
SCPE	0.8847 ± 0.0304^##^	0.9619 ± 0.01388^##^
F value	533.319	1,268.604
*P* value	0.001	0.001

Note: ^**^ *p* < 0.01, compared with Control group; ^##^ *p* < 0.01, compared with Model Group.

#### 3.5.2 The effects of SCPE on functional plasticity of hippocampal synapses in Dgalactose model mice

Finally, we assessed PSD95 and AMPA protein expression to observe the effect of SCPE on synaptic functional plasticity. Compared with the control group, the expression levels of PSD95 and AMPA proteins in the hippocampus of the model group mice were significantly decreased (*p* < 0.01). Compared with the Model group, the expressions of PSD95 and AMPA proteins in the hippocampus of the Postive group and SCPE group were increased (*p* < 0.01) (Figures 5I–K; Table 9).

**TABLE 9:** Protein expression of PSD95 and AMPA in mouse hippocampus (mean ± SD, *n* = 3).

Group	PSD95/GAPDH	AMPA/GAPDH
Control	1.000 ± 0.0000	1.000 ± 0.0000
Model	0.4400 ± 0.0085^**^	0.2594 ± 0.0921^**^
Positive	1.3130 ± 0.0459^##^	0.7788 ± 0.0053^##^
SCPE	1.0934 ± 0.0123^##^	0.8860 ± 0.0005^##^
F value	710.459	150.991
*P* value	0.001	0.001

Note: ^**^ *p* < 0.01, compared with Control group; ^##^ *p* < 0.01, compared with Model Group.

## 4 Discussion

The cognitive dysfunction model replicated by Dgalactose accelerates aging in mice by increasing oxidative stress damage and abnormal phosphorylation in the body, eventually causing neuronal damage and synaptic failure in the hippocampus of mice (Lu et al., 2010), resulting in the deterioration of learning and memory ability (Xian et al., 2011). We employ Morris water maze and novel object recognition experiments to test learned cognitive abilities. The Morris water maze experiment is widely used in the study of brain learning and memory mechanisms, which can evaluate the ability of mice to learn to navigate to specific spatial locations in a relatively large spatial environment (Vorhees and Williams, 2006). In this experiment, SCPE decreased the latency of Model group mice, and increased the number of Model group mice crossing the platform and the residence time of the target quadrant. Novel object recognition experiments uses the principle that animals have an innate tendency to explore new objects to examine the ability of animals to learn and explore. In this experiment, SCPE can significantly improve the learning and exploration ability of Model group mice. Combined with the results of behavioral experiments, SCPE can improve the learning and memory impairment of model mice. Through the observation of hippocampal tissue morphology, we found that SCPE can also repair the damaged neuronal state in model mice.

SCPE is widely used in traditional Chinese medicine to treat AD, but its effective chemical components and its mechanism of action are still unclear. UPLC-LTQ-Orbitrap-MS combined with network pharmacology method was used to study the active components of SCPE and its anti-AD mechanism. Ultra performance liquid chromatography-mass spectrometry (UPLC-MS) technology has high-efficiency liquid chromatography separation characteristics and strong identification capabilities of mass spectrometry, and has been widely used in the qualitative analysis of chemical components in traditional Chinese medicine. We optimized UPLC-LTQ-Orbitrap-MS processing method to obtain more chemical composition. The results showed that SCPE contained 9 blood components, including 2 phenolic components, four oligosaccharide ester components, 2 xanthones components, and 1 saponin component. As far as we know, this is the first study on UPLC-LTQ-Orbitrap-MS combined with network pharmacology analysis to explain the chemical signature of SCPE’s anti-AD effect.

Network pharmacology analysis can reveal the relationship between chemical components and targets/pathways based on component analysis, and establish “compound-target-pathway” to identify components and mechanisms that perform specific pharmacological activities. PPI network analysis predicts that 15 targets including *ALB*, *EGFR*, *TNF*, *MMP9*, *ESR1*, *STAT3*, *APP*, *MMP2*, *HSP90AA1*, *HRAS*, *MAPK14*, *TLR4*, *KDR*, *MAPK8*, *CYP19A1* are key targets for SCPE in the treatment of AD. Among these key targets in the network, *EGFR* signaling inhibits synaptic target selection by type II neurons, reducing synaptic plasticity (Naylor and DiAntonio, 2012). Matrix metalloproteinases (MMPs) are a family of Zn^2+^-dependent endopeptidases that regulate tissue homeostasis in the adult brain and are now widely used in central nervous system diseases (Mott and Werb, 2004; Rivera et al., 2010). *MMP2* and *MMP9* can rapidly regulate dendritic spine morphology and contribute to neuronal activity-dependent structural plasticity of dendritic spines (Szepesi et al., 2013). Among them, both functional and structural plasticity induced by *MMP9* are dependent on signaling through integrins containing the β1 subunit and are associated with integrin-dependent phosphorylation of the actin depolymerizing factor cofilin within dendritic spines (Huntley, 2012). *ESR1* is the estrogen receptor alpha (ERα) gene. As a nuclear receptor that binds to estrogen, ERα participates in neuroprotection in the brain (Jelks et al., 2007) and regulates hippocampal synaptic plasticity (Long et al., 2012; Phan et al., 2015). Estradiol-induced increases in midcapital spines were observed in ERα-expressing mice, and estradiol reduced AMPA receptor-mediated miniature excitatory postsynaptic current amplitudes and membrane depolarization in CA1 pyramidal neurons. And it rapidly increasing NMDA-induced synaptic long-term depression (LTD.) in CA1, estrogen via ERα activation rapidly and nongenomically increases the formation of silent or immature synapses within CA1 hippocampus, promoting plasticity and promoting learning. *STAT3* is involved in regulating synaptic transmission and plasticity through genomic mechanisms on the one hand, and affects synaptic transmission by altering the polarity of cerebellar synaptic plasticity on the other hand. Knock out *STAT3* increases AMPA receptor-mediated mini-excitatory postsynaptic current amplitude and attenuates mini-inhibitory postsynaptic current frequency (Han et al., 2021). *APP*, the first protein associated with sporadic AD, is mainly expressed in excitatory neurons in the adult cerebral cortex and can autonomously regulate synaptic plasticity and neuronal excitability in mature excitatory neurons in the cerebral cortex (Lee S. H. et al., 2020). Mutant *APP* had the opposite effect, which reduces the dendritic protein MAP2, dendritic spine levels, the synapsin synaptophysin, and PSD95, resulting in synaptic damage (Manczak et al., 2018). *HRas* are involved in synaptic transmission and plasticity. Knockdown of HRas contributes to the upregulation of NMDA synaptic responses and synaptic long-term potentiation (LTP), modulating the number of surface AMPA receptors. Knockdown of *HRas* also increased AMPA receptors during synaptic potentiation (CaMKII) and decreased synaptic plasticity during synaptic scaling (CDK5) (Manabe et al., 2000). Key targets in the network are closely linked to estrogen signaling, serotonergic synapses, dopaminergic synapses, MAPK signaling, and regulation of the actin cytoskeleton, and these results focus on estrogen and synapses plasticity. These results focus on estrogen and synaptic plasticity, providing preliminary evidence for the pharmacological mechanism of SPEG in AD treatment. Interestingly, in our experiments, we found that the SCPE group had similar anti-AD effects to the estrogen Positive group.

Epidemiological studies suggest that the risk of AD increases with loss of age-related steroid hormones and that AD is more prevalent in postmenopausal women than in age-matched men (Viña and Lloret, 2010). This is because estrogen is produced in the brain through two different pathways. On the one hand, estrogen in the brain is secreted by the corpus luteum and follicle in the ovary, and is transmitted to the blood-brain barrier through the systemic circulation to replenish the estrogen level in the brain. On the other hand, using androgens as synthetic substrates through aromatase (CYP19) synthesis in the ovary, neuronal cells in the brain can synthesize trace amounts of brain-derived estrogens through CYP19 regulation of cholesterol present in neurons through various channels. The dramatic decline in ovarian estrogen and progesterone after menopause in women, while estrogen in the male brain can be produced by aromatase-catalyzed testosterone or *de novo* synthesis in neurons or glial cells is thought to account for the increased susceptibility to AD in women (Kong et al., 2019). Estrogen is known to have a protective effect on the brain, and estrogen can increase neurogenesis in various regions of the brain, for example increasing newly generated neurons in the hippocampus contributes to region-specific learning and memory (Galea et al., 2013). Estrogen deficiency in the brain increases the risk of AD, and estrogen loss during menopause may contribute to brain metabolic deficits in AD (Long et al., 2012). Decreased circulating levels of estradiol and testosterone and down-regulation of brain aromatase expression are also prevalent in the brains of AD patients (Callahan et al., 2001; Ishunina et al., 2005). Through experiments, we found that SCPE can increase the content of E2 in blood, and can effectively upregulate the expression of *CYP19 mRNA* in the hippocampus of mice, thereby increasing brain-derived estrogen. After supplementing estradiol, it can significantly increase the estrogen effect, but it cannot mediate *CYP19 mRNA* expression increases brain-derived estrogen, suggesting that SCPE has an estrogen-like effect.

The occurrence of estrogenic effects in brain regions is not only affected by E2 levels, but also related to the expression of estrogen receptors. As a nuclear receptor that binds to estrogen, ERα is involved in neuroprotective effects in the brain (Rune et al., 2002), and it is highly expressed in the hippocampus, so it is speculated that effectively increasing the expression of ERα will help to enhance the estrogen effect in the hippocampus. The experimental results show that SCPE can enhance the expression of ERα. Combined with the results of behavior, histomorphology, E2 content and *CYP19 mRNA*, SCPE may play a neuroprotective role by enhancing the effect of estrogen in the brain. The main ways include: a.SCPE can increase the level of E2 in the body; b.Increase the level of aromatase in the brain, and promote the synthesis of brain-derived E2 by hippocampal neurons; play” E2-ERα" pathway dominance effect.

E2 selectively activates ERα receptors in the brain and releases brain-derived BDNF to alter the function and structure of excitatory synapses. As a releasable neurotrophic factor, BDNF can stimulate RhoA to regulate the cofilin pathway, and can also activate Rac and Cdc42 in addition to Rho. As a releasable neurotrophic factor, BDNF can stimulate RhoA to regulate the cofilin pathway, and can also activate Rac and Cdc42 in addition to Rho to activate the effector protein PAK, thereby coordinating the activities of cofilin and procilin. Ultimately promote actin accumulation to stabilize dendritic spine structure and improve synaptic structural plasticity (Spencer et al., 2008; Kramár et al., 2009; Kwon and Sabatini, 2011). BDNF can also enhance synaptic function by increasing the expression of PSD95 and the functional receptor AMPA by activating specific pathways. The experimental results are as expected, SCPE can increase the release of brain-derived BDNF by enhancing the estrogenic effect of E2-selective activation of ERα receptors in the brain, thereby changing the function and structure of excitatory synapses.

The study found that both estrogen and BDNF have important effects on synaptic plasticity. Combined with network pharmacology, it is speculated that SCPE can achieve anti-AD effect by increasing the effect of estrogen in the brain and further regulating synaptic plasticity by brain-derived BDNF. Synaptic plasticity can be divided into synaptic structural plasticity and synaptic functional plasticity, both of which jointly determine the normal biological effects of the central nervous system and the construction of the related learning and memory mechanism in the hippocampus (Vose and Stanton, 2017). The changes in synaptic structural plasticity are mainly related to the structural stability of dendritic spines. Dendritic spines are the main components of the excitatory postsynaptic membrane of neurons, and their functions include receiving signals from the previous neuron and expanding the surface area of the postsynaptic membrane for receiving signals to carry postsynaptic compact proteins and expression of its functional receptors (Yoshihara et al., 2009; Roberts et al., 2010). The cytoskeleton of dendritic spines is composed of actin (actin), and monomeric globular protein (G-actin) and multimeric filamentous protein (F-actin) are the two existing forms of actin. The protein content ratio of F-actin/G-actin reflects the stability of the dendritic spine cytoskeleton. If the ratio increases in the direction of G-actin, it indicates that the cytoskeleton is in a state of depolymerization, which indicates the damage of synaptic plasticity; The increase in the direction of F-actin indicates that the cytoskeleton is in a state of aggregation, marking the stability of the dendritic spine cytoskeleton and improving synaptic plasticity. Stabilization of dendritic spine skeleton actin is also associated with cofilin phosphorylation. As an active protein that can cut dynamic actin filaments (Mizuno, 2013; Feng et al., 2019), cofilin increases phosphorylation of cofilin under the action of E2 (Feng et al., 2019), thereby stabilizing actin filaments and promoting actin polymerization (Hong et al., 2021). The experimental results show that SCPE can increase the ratio of F-actin/G-actin, reduce the level of cofilin protein, increase the level of P-cofilin protein, effectively improve the depolymerization of cytoskeletal proteins in the hippocampus of Dgalactose model mice, and increase the plasticity of synaptic structure.

**FIGURE 6:**
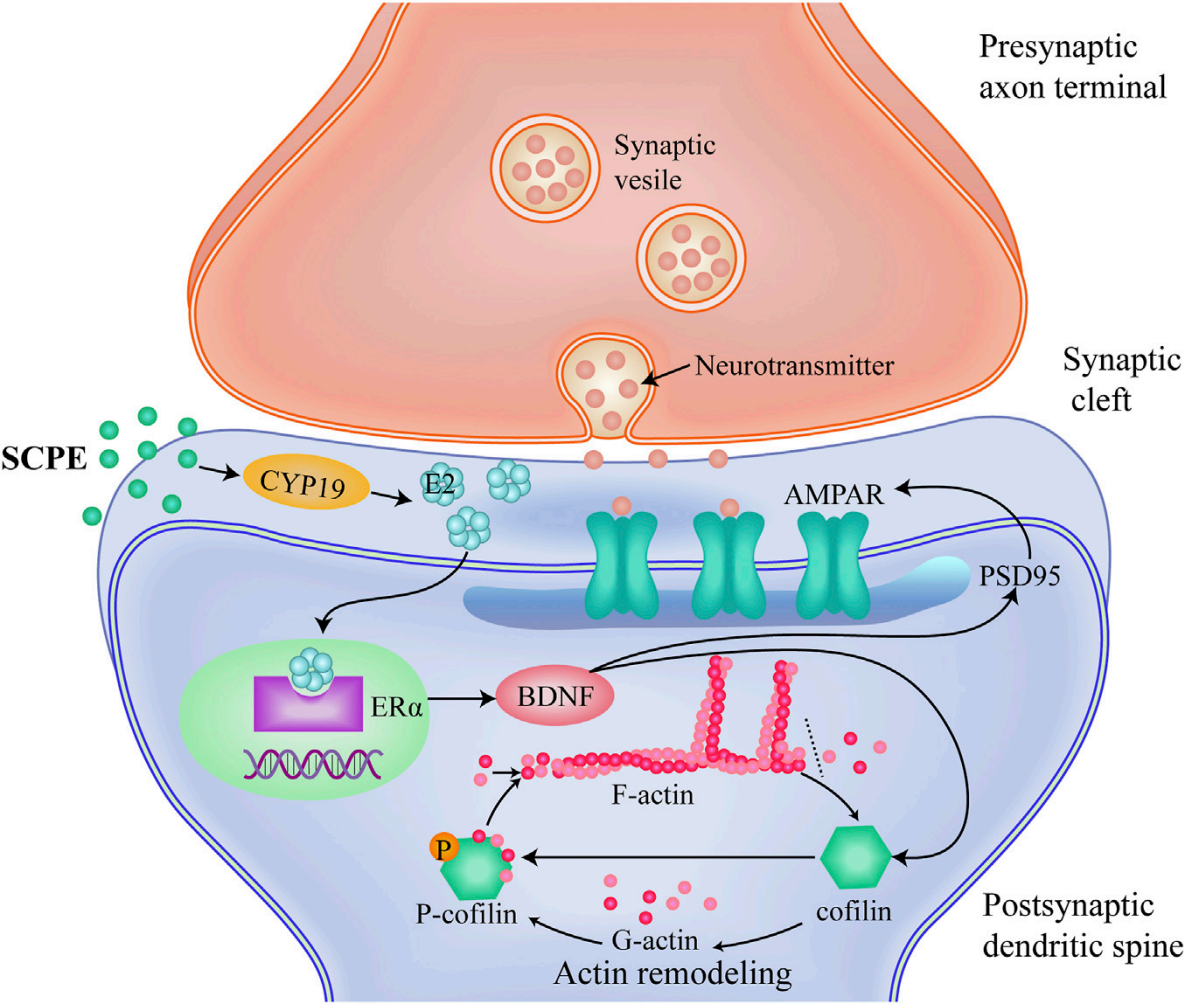
A proposed mechanism which SCPE could be a potential treatment for AD.SCPE increases the levels of E2 and CYP19, promotes the synthesis of brain-derived E2 in hippocampal neurons.And then SCPE induces the increased expression of ERα in the brain to exert estrogenic effects.Estrogenic effects increase the release of brain-derived BDNF and alter the function and structure of excitatory synapses.Furthermore, SCPE interacts with multiple proteins at synapses. It phosphorylates cofilin to stabilize actin filaments and promotes actin polymerization. Actin polymerization results in dendritic spine enlargement, and lateral movement of AMPARs into the synapse.These interactions integrate upstream signals into dynamic changes in the actin cytoskeleton that work to regulate synaptic plasticity. SCPE finally improves synaptic plasticity by increasing the effect of estrogen to achieve the purpose of treating AD.

Changes in synaptic functional plasticity are mainly associated with PSD95 and its binding AMPA functional receptors (Xu, 2011; Jeyifous et al., 2016). PSD95 is the most abundant and functional scaffold protein distributed on the excitatory synaptic membrane of the hippocampus (Xu, 2011). The dendritic spines protruding from the dendrite surface of neurons are wrapped by PSD95, which is the binding site for the expression and anchoring of functional receptors. It is a landmark protein related to synaptic function, and its representative functional receptor AMPA (Jeyifous et al., 2016; Xiao et al., 2016; Zaręba-Kozioł et al., 2018). The high expression of PSD95 can increase the binding area and binding capacity of AMPA, and improve synaptic function (Nusser, 2000). AMPA, as an ionotropic glutamate receptor, mainly mediates the transmission of excitatory synaptic neurotransmitters, and is involved in the change and maintenance of LTP. Synaptic functional plasticity is mediated by ERα-BDNF. BDNF induces direct translation of AMPA receptor protein and subsequent receptor insertion, which has a significant regulatory effect on AMPA expression and membrane transport, resulting in enhanced synaptic plasticity (Caldeira et al., 2007; Fortin et al., 2012). BDNF also induces calmodulin-dependent protein kinase (CAMKK) to activate the mammalian target of rapamycin (mTOR), which binds synthetic AMPA to PSD95 via the adaptor protein transmembrane AMPA regulatory protein (TARPS) to form a complex that inhibits the formation of a complex, and retention of receptors at the contact site plays a key role (Fortin et al., 2012). In this experiment, on the premise that SCPE increases the estrogen effect in the brain by mediating ERα-BDNF, it is found that SCPE up-regulates the expression of PSD95 and the expression of functional AMPA receptors in postsynaptic membranes, ensuring the synapses functional plasticity. In a word, our findings show that SCPE may improve synaptic plasticity by increasing estrogenic effects to treat AD (Figure 6).

## 5 Conclusion

In conclusion, this study used UPLC-LTQ-Orbitrap-MS to conduct a comprehensive chromatographic analysis of SCPE-containing rat serum and identified 9 blood components. And combined with the network pharmacology method to study the mechanism of SCPE on AD, a total of 15 key targets of SCPE in the treatment of AD were screened out. These key targets are closely related to estrogen signaling pathway, serotonergic synapse, dopaminergic synapse, regulation of actin cytoskeleton and other pathways, which are unprecedentedly explored by SCPE. And provide a reference for determining the pharmacodynamic material basis of SCPE in the treatment of AD. The results obtained from the above experiments continue to further explore the mechanism of action of SCPE in the treatment of AD. It is expounded that SCPE can improve the structural plasticity and functional plasticity of hippocampal synapses by promoting the synthesis of brain-derived E2 in hippocampal neurons, inducing the increase of ERα expression in the brain, and enhancing the effect of estrogen in the brain, thereby exerting a neuroprotective mechanism. This provides a basis for further mechanism studies of SCPE, and also provides evidence for the development of traditional Chinese medicine SCPE as a potential treatment for AD patients. However, this study also has some limitations. It has not been clarified whether SCPE cooperates with multiple ER receptors to exert neuroprotective effects. Next, we will set up different types of estrogen blockers combined with gene knockdown and other methods to supplement the neuroprotective effects of SCPE. And through multi-omics combined technology to prove the mechanism from multiple perspectives. It will be the focus of our future research.

## Data Availability

The original contributions presented in the study are included in the article/Supplementary Materials, further inquiries can be directed to the corresponding authors.

## Supplementary material

Supplementary Figure S1 The trajectory of the mouse to find the platform.

## Abbreviations

SCPE, Sagacious Confucius’ Pillow Elixir; KM, Kun Ming.
