# Supplementary figures and images for: Sagacious confucius’ pillow elixir ameliorates Dgalactose induced cognitive injury in mice via estrogenic effects and synaptic plasticity
Source: Front Pharmacol. 2022 Sep 28;13:971385. doi: 10.3389/fphar.2022.971385 (PMC9555387; doi:10.3389/fphar.2022.971385)

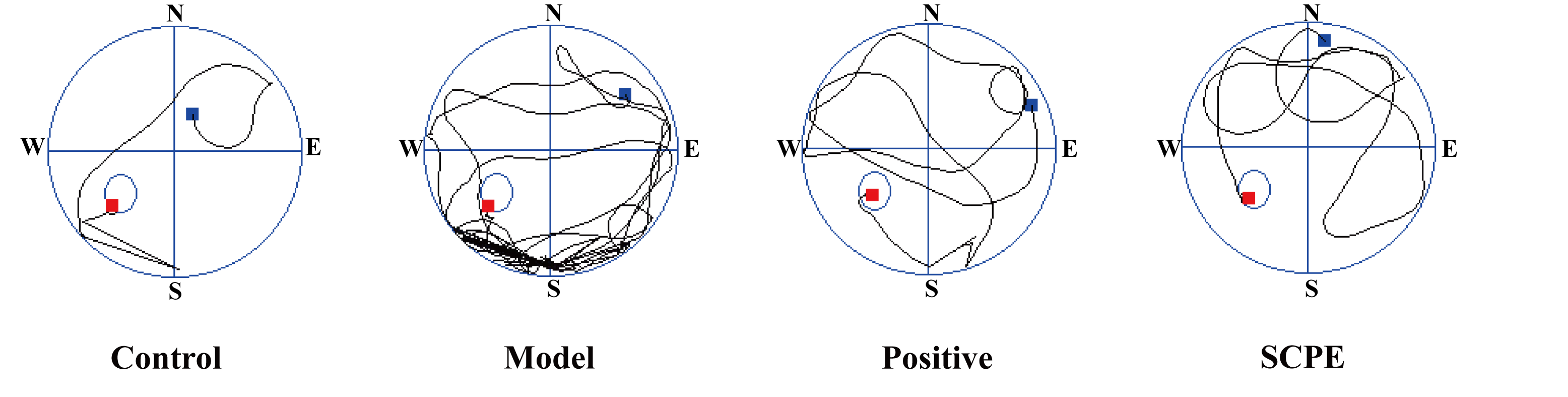

Supplement: Supplementary file 1 [file Image1.tif]
